# Reviewers for 2025

**DOI:** 10.4314/gmj.v60i1.1

**Published:** 2026-03

Reviewers of our journal's manuscripts contribute immensely to the quality and standards of the papers we publish. On behalf of the Editorial Board, we thank all the reviewers who generously contributed their time, expertise, and thoughtful critique to our peer review process when called upon in 2025.

Your commitment to rigorous evaluations and constructive feedback remains at the heart of how we ensure the scientific integrity, quality, and credibility of the work we publish. We greatly appreciate your contribution to the advancement of scholarship and to the assistance authors receive in developing their works. We acknowledge your efforts, given the toll imposed on your busy schedules.

The Ghana Medical Journal is grateful to the following reviewers for their voluntary service in 2025.

**Table:** 

Name	Country
Ababio, Grace	Ghana
Abbeyquaye, Emmanuel	Ghana
Aborisade, Adetayo	Nigeria
Abrahams, Afua	Ghana
Achampong, Emmanuel	Ghana
Adeko, Oluseun	Nigeria
Adeyemi, Michael	Nigeria
Adjei, Ernest	Ghana
Agadaga, Ifeakachukwu	Nigeria
Ago, Boniface	Nigeria
Agyeman-Yeboah, Joana	Ghana
Ajayi, Isaac	Ghana
Akase, Iorhen	Nigeria
Akinbodewa, Akinwumi	Nigeria
Akintunde, Adeseye	Nigeria
Akowuah, Jones Asafo	Ghana
Akpakli, Elizabeth	S Africa
Akpalu, Josephine	Ghana
Ali, Sajjad	Pakistan
Amoah, Kwadwo	Ghana
Amoako, Thomas	Ghana
Amoaku, Winfried	United. Kingdom
Amoh, Michael	Ghana
Ampofo, Eugene	Ghana
Amponsah, Emmanuel	Ghana
Aniteye, Ernest	Ghana
Ankrah, Daniel	Ghana
Ansong, Daniel	Ghana
Antwi, Sampson	Ghana
Anyanechi, Charles	Nigeria
Appeadu-Mensah, William	Ghana
Appiah-Brempong, Emmanuel	Ghana
Appiah-Opong, Regina	Ghana
Arthur, Joshua	Ghana
Asuquo, Marcus	Nigeria
Awindaogo, Felix	Ghana
Awua, Adolf Kofi	Ghana
Ayi, Irene	Ghana
Balamurali, Adhithya Jeevan	India
Bandoh, Salome	Ghana
Bayoumi, Nader	Egypt
Bayuo, Jonathan	Ghana
Bediako-Bowan, Antoinette	Ghana
Bello, Ajediran	Nigeria
Bello, Bashir	Nigeria
Beyuo, Vera	Ghana
Boadu, Ama	Ghana
Boamah, Martin	Ghana
Boateng, Laurene	Ghana
Clegg-Lamptey, Joe-Nat	Ghana
Dakubo, Jonathan	Ghana
Darko, Kwame	Ghana
Dey, Dzifa	Ghana
Dogbe, Yaunuick	Ghana
Djagbletey, Robert	Ghana
Donkor, Peter	Ghana
Dovlo, Dela	Rwanda
Duah, Amoako	Ghana
Duodu, Fiifi	Ghana
Ebeigbe, Jennifer	Nigeria
Ekremet, Kwame	Ghana
Entsua-Mensah, Kow	Ghana
Enweronu-Laryea, Christabel	Ghana
Esimai, Peju	Nigeria
Etwire, Victor	Ghana
Fiagbe, Delali	Ghana
Forson, Audrey	Ghana
Ganyaglo, Gabriel	Ghana
Geethanath, Sairam	United States
Gyan, Ben	Ghana
Gyan, Desrie	Ghana
Gyedu, Adam	Ghana
Ibadi, Mohammed	Iraq
Issa, Amudalat	Nigeria
Jimah, Bashiru	Ghana
Kenu, Ernest	Ghana
Kokuro, Collins	Ghana
Koram, Kwadwo	Ghana
Kwakyi, Edward	Ghana
Kwame-Aryee, Robert	Ghana
Kyei, Samuel	Ghana
Lartey, Margaret	Ghana
Lawal, Aisha	Nigeria
Lyrawati, Diana	Indonesia
Mensah, Kofi	Ghana
Mensah, Yaw	Ghana
Michael, Godpower	Nigeria
Muhammad, Abubakar Sadiq	Nigeria
Mutalub, Yahkub	Nigeria
Nguah, Samuel	Ghana
Nkanga, Elizabeth	Nigeria
Nkrumah, Kofi	Ghana
Nkrumah-Mills, John	Ghana
Nkyekyer, Kobina	Ghana
Nsaful, Josephine	Ghana
Nsaful, Kwesi	Ghana
Obeng Adjei, George	Ghana
Obuobie, Kofi	United Kingdom
Ofori-Adjei, David	Ghana
Ohene-Darkoh, Charles	Canada
Opoku, Juliet	Ghana
Osinga, Joris A. J.	Netherlands
Osundina, Morenike	Nigeria
Oyewole, Olufemi	Nigeria
Quaye, Isaac	Ghana
Sarfo, Fred	Ghana
Sefogah, Promise	Ghana
Seneadza, Nana Ayegua Hagan	Ghana
Tachi, Kenneth	Ghana
Tagoe, Lily	Ghana
Taherkhani, Reza	Iran
Tannor, Elliot	Ghana
Wagatsuma, Yukiko	Japan
Wiafe, Yaw	Ghana
Yakubu, Rafiuk	Ghana
Yawson, Alfred	Ghana
Zyluk, Andrzej	Poland

